# The feasibility and accuracy of machine learning in improving safety and efficiency of thrombolysis for patients with stroke: Literature review and proposed improvements

**DOI:** 10.3389/fneur.2022.934929

**Published:** 2022-10-20

**Authors:** Huiling Shao, Xiangyan Chen, Qilin Ma, Zhiyu Shao, Heng Du, Lawrence Wing Chi Chan

**Affiliations:** ^1^Department of Health Technology and Informatics, The Hong Kong Polytechnic University, Hong Kong, Hong Kong SAR, China; ^2^Department of Neurology, The First Affiliated Hospital of Xiamen University, Xiamen, China

**Keywords:** acute ischemic stroke, neuroimaging, machine learning, thrombolysis, clinical decision support tool, penumbra, translational medicine

## Abstract

In the treatment of ischemic stroke, timely and efficient recanalization of occluded brain arteries can successfully salvage the ischemic brain. Thrombolysis is the first-line treatment for ischemic stroke. Machine learning models have the potential to select patients who could benefit the most from thrombolysis. In this study, we identified 29 related previous machine learning models, reviewed the models on the accuracy and feasibility, and proposed corresponding improvements. Regarding accuracy, lack of long-term outcome, treatment option consideration, and advanced radiological features were found in many previous studies in terms of model conceptualization. Regarding interpretability, most of the previous models chose restrictive models for high interpretability and did not mention processing time consideration. In the future, model conceptualization could be improved based on comprehensive neurological domain knowledge and feasibility needs to be achieved by elaborate computer science algorithms to increase the interpretability of flexible algorithms and shorten the processing time of the pipeline interpreting medical images.

## 1. Introduction

Stroke is the most common neurological disease (1) which can be defined as an acute central nervous system injury with an abrupt onset. Stroke is the third leading cause of death and chronic disability globally (1). As a leading cause of adult disability, up to 74% of stroke survivors are dependent on activities of daily living (2), which causes a huge burden to society, both in finance and human resources. Among different types of stroke, ischemic stroke is the most common, accounting for 87% compared to hemorrhagic stroke (3). The etiology of ischemic stroke is the obstruction of cerebral arteries due to multiple reasons, which could be classified as five subtypes according to the Trial of Org 10172 in Acute Stroke Treatment (TOAST) criteria (4). Because of the TOAST mechanisms, the decreased blood perfusion to the brain leads to ischemic stroke.

In the treatment of ischemic stroke, timely and efficient recanalization of occluded brain arteries can successfully salvage ischemic brain (5). An intravenous (IV) injection of recombinant tissue plasminogen activator(rtPA)—also called alteplase—is the first-line treatment for ischemic stroke (6). For patients with acute ischemic stroke, a prompt treatment with thrombolytic drugs could restore blood flow before major brain damage has occurred and greatly improve short-term and long-term recovery after stroke (7), as a result largely reducing the burden stroke brings to the society.

In most cases, IV thrombolysis therapy is subject to the latest guidelines. The guidelines are drawn up based on large quantities of clinical evidence, therefore, the proposed eligibility and dosage consideration for thrombolysis treatment should normally be safe and efficient for most of the patients. However, in real clinical practice, still, several patients present unpredictable outcomes after the IV thrombolysis treatment, including symptomatic hemorrhage (13% among patients receiving rtPA) (8) and failed recanalization (37% among patients receiving rtPA) (9), suggesting that a more accurate patient-tailored clinical decision support tool based on guidelines to improve IV thrombolysis safety and efficiency is needed.

The literature on machine learning models to assist in stroke thrombolysis has yet to be systematically synthesized and assessed for accuracy and feasibility. Most of the existing reviews have focused on the accuracy of clinical outcome prediction models for patients with acute ischemic stroke, albeit not focused on thrombolysis specifically (10). Some reviews focusing on thrombolysis did not analyze the feasibility of these models in hyperacute clinical stroke settings (11).

To address this gap, we reviewed the literature on the accuracy and feasibility of machine learning models to assist in stroke thrombolysis. This review aims to address the following research questions: (1) What criteria should a machine learning model meet in order to be accurate and feasible in real clinical practice? (2) Have previous machine learning models met these criteria? (3) What improvements could be proposed to increase the accuracy and feasibility of previous models?

## 2. Search methods and results

**Figure 1:**
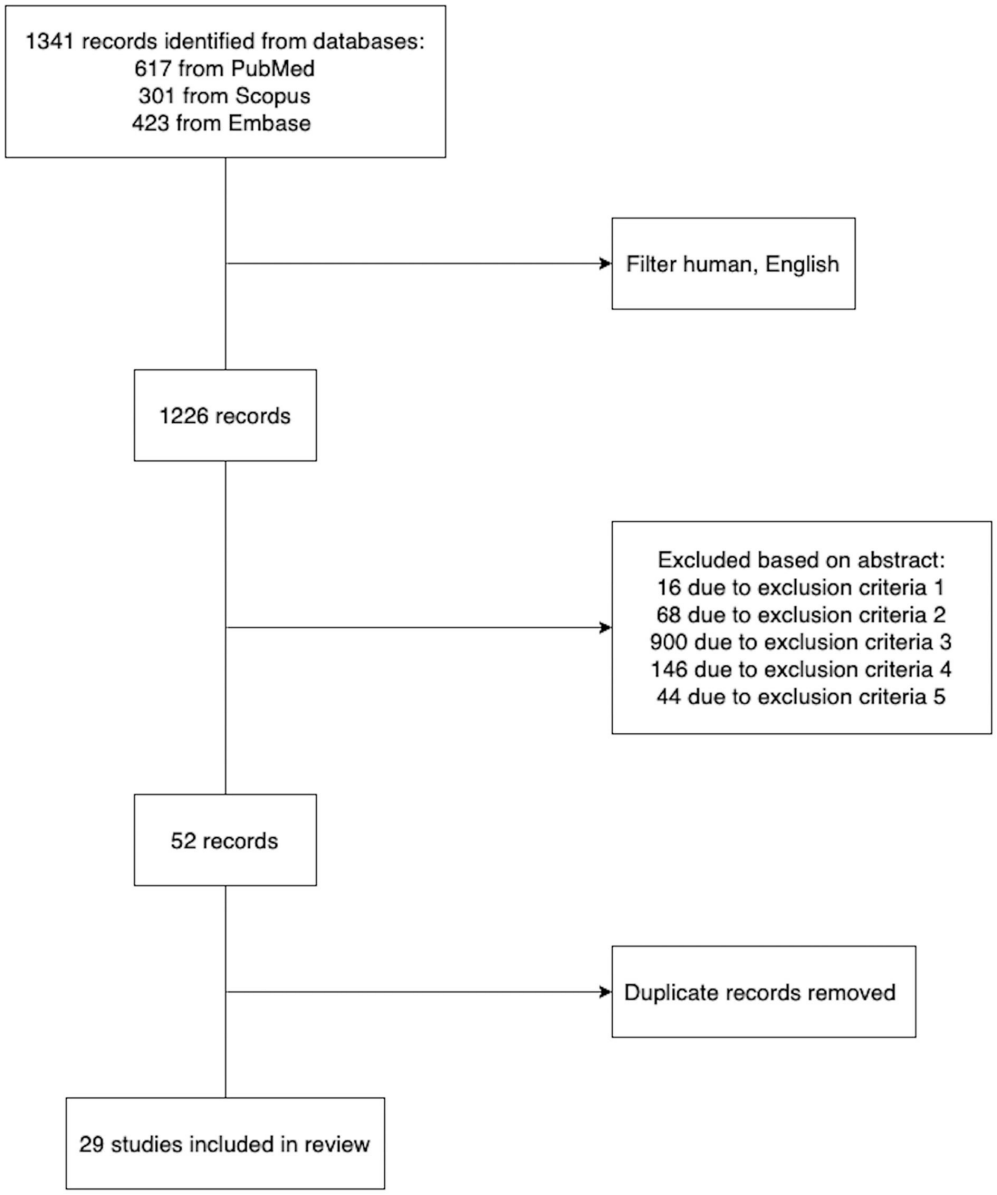
Selection of studies. The five exclusion criteria were explained in detail in Section 2.

PubMed, Embase, and Scopus (inception to July 2022) were searched to identify studies developing machine learning models to assist in deciding the personalized safety and efficiency of thrombolysis therapy. We used Medical Subject Headings (MeSH) terms in multiple combinations, including stroke thrombolysis/machine learning and stroke thrombolysis/prediction model, to retrieve papers. The search was limited to human studies with English restrictions applied. The inclusion and exclusion criteria of each study were reviewed. We excluded studies where: (1) The full paper was not available. (2) The paper presented review findings instead of original research. (3) Participants enrolled did not receive thrombolysis therapy. (4) The objective was to infer the association between thrombolysis clinical outcome and biomarkers rather than predict the outcome accurately. (5) The prediction model can only be applied to patients with a specific subtype of ischemic stroke. In the end, we retrieved 29 representative research papers (Figure 1). The detailed information of the representative papers is presented in Supplementary Table 1.

## 3. Feasibility and accuracy

**Figure 2:**
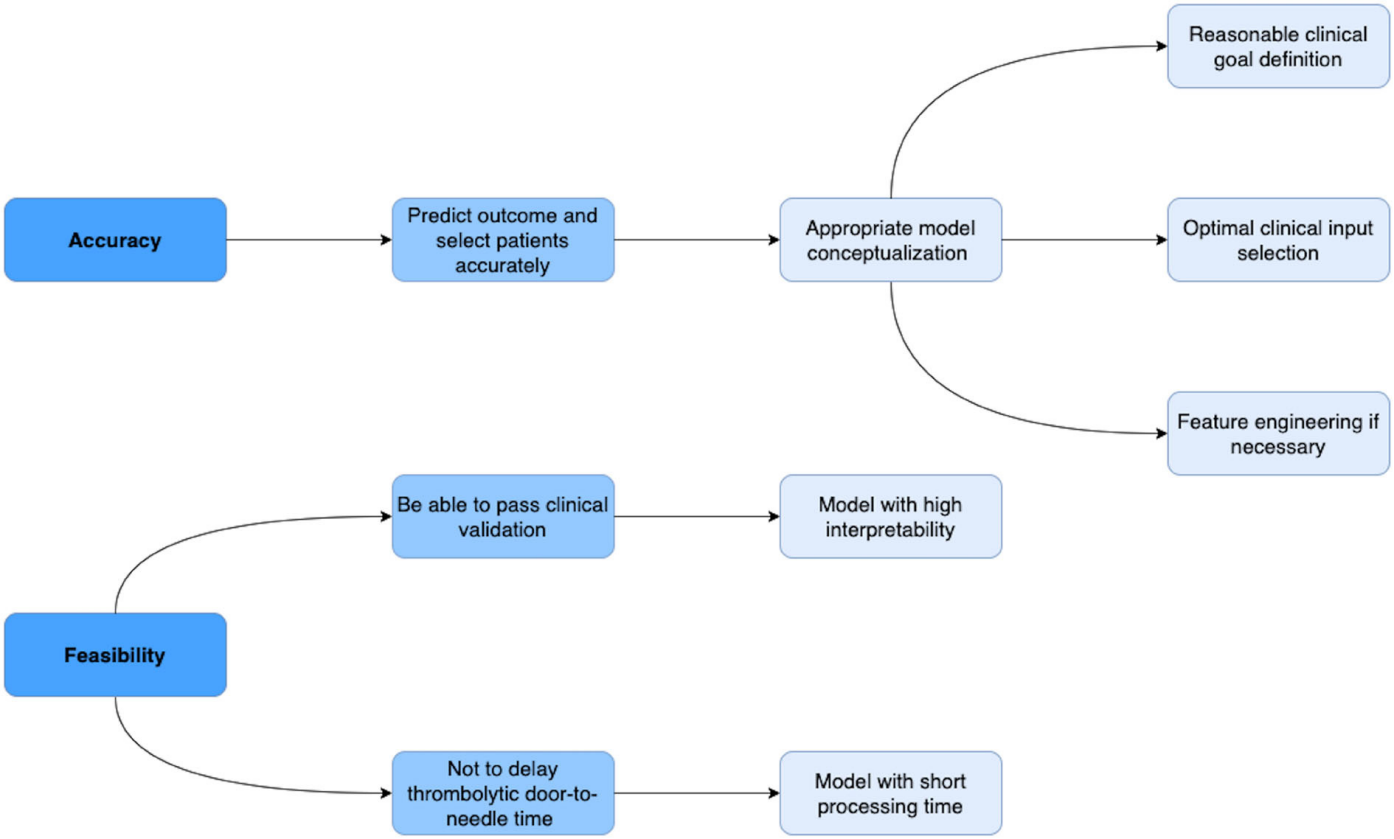
Blueprint of the criteria a machine learning model should meet in order to be accurate and feasible in assisting thrombolysis therapy. In terms of accuracy, a model should be able to predict risks and benefits and select target patients who could benefit the most from thrombolysis therapy with high accuracy. The accuracy of the model largely depends on the conceptualization, which consists of reasonable clinical goal definition, optimal clinical input selection, and appropriate feature engineering if necessary. In terms of feasibility, in order to pass the clinical validation, a model should have high interpretability; in order not to delay the door-to-needle time, the model needs to calculate the output in a short time.

Machine learning models are computer algorithms developed to imitate the human learning process. The training of machine learning models consists of a phase where models improve their accuracy by discovering patterns and associations within huge datasets. This training principle allows machine learning models to generate satisfactory results, especially in evidence-based practices, such as medicine. Given the fact that in real clinical practice, thrombolysis therapy respecting guideline has a relatively low percentage of successful recanalization, some experienced clinicians might decide the eligibility and dosage for certain patients based on their own clinical experiences (12). According to Dr. Patrick D. Lyden's review article: The decision to use thrombolytic therapy is—among the most difficult treatment decisions in medicine, given the risks involved and the compressed time frame available (13). Machine learning models with high accuracy and feasibility have the potential to acquire clinical experience from real world large datasets of patients undergoing thrombolysis and assist in improving the safety and efficiency of IV thrombolysis therapy. Figure 2 provides a blueprint of the criteria a machine learning model should meet in order to be accurate and feasible in assisting thrombolysis therapy.

In the thrombolysis setting, a model is conceptualized in order to (1) predict risks and benefits, which can be considered respectively as poststroke symptomatic intracerebral hemorrhage and long-term outcome, as well as (2) select patients with stroke who could benefit the most from thrombolysis therapy in high accuracy. The accuracy of a machine learning model largely depends on appropriate model conceptualization. The widely accepted formal definition of machine learning as stated by field pioneer Mitchell (14): A computer program is said to learn from experience E with respect to some class of tasks T and performance measure P if its performance at tasks in T, as measured by P, improves with experience E. When conceptualizing a machine learning model, a clinical data scientist generally answers two questions: what the goal of the machine learning model is (clinical goal definition) and what clinical variables capture the experience required to achieve the goal (clinical feature selection). In some cases, if raw clinical features are not able to capture the semantics that the human brain understands from the dataset, such as some radiological features representing penumbra and proximal/distal arterial occlusion information, feature engineering is required to create/extract features using domain knowledge. Feature Engineering can use data mining techniques to preserve these semantics and help machine learning algorithms to understand data and determine patterns that can improve the performance of machine learning algorithms.

Besides accuracy, feasibility is also an important, however often ignored, factor to consider when developing clinical machine learning models. We here identify two factors that will hinder the implementation of models in the thrombolysis setting: the interpretability and processing time of the model. The interpretability of the algorithm is critical since all clinical decision support tools must go through clinical trials to be approved by the local authority before being used in real clinical practice. The interpretability of the algorithm allows telling which predictors the algorithm leverages as important factors to be considered when predicting the clinical outcome or deciding the thrombolysis eligibility. These predictors then need to be confirmed related to the clinical outcome of patients going through thrombolysis by previous clinical trials or following clinical trials in case the algorithm generates new features during training. Furthermore, given the fact that human nervous tissue is rapidly lost as stroke progress and longer thrombolytic door-to-needle time is associated with higher mortality (15), the processing time of a thrombolytic clinical decision support tool should be measured and the tool should be able to produce the outcome shortly so as not to delay the treatment.

In the following sections, we are going to analyze previous studies based on these criteria and propose improvements to increase the accuracy and feasibility of previous models.

## 4. Clinical goal definition

In a thrombolysis setting, a model is conceptualized in order to (1) predict risks and benefits, as well as (2) select patients with stroke who could benefit most from thrombolysis therapy in high accuracy. Previous studies achieved the goal by developing an efficiency and safety prediction model. Among all the literature reviewed, 11 developed models with the objective to assess thrombolysis efficiency and 16 developed models with the objective to assess thrombolysis safety. Only two assessed both efficiency and safety. The clinical outputs of models predicting safety are all poststroke symptomatic intracerebral hemorrhage (16–33) while the clinical outputs of models predicting efficiency vary: the most common is the 3-month modified Rankin Scale(mRS) (25, 34–38). Huang et al. (39) used an even longer 6-month mRS. Saposnik et al. (22) leveraged a composite 3-month outcome of mRS, National Institutes of Health Stroke Scale (NIHSS), and Barthel index and Glasgow Outcome Scale score. Some models provided both predictions on early clinical outcomes and a long-term 3-month mRS (40, 41). A recent model in 2021 (42) predicted the final infarct volumes for patients after thrombolysis therapy. Zhu et al. (43) only predicted 1-h NIHSS after thrombolysis. The early outcome advantage of thrombolysis does not necessarily persist during long-term follow-up. To provide a comprehensive thrombolysis efficiency assessment, both early and long-term outcome predictions are required.

Most of the models were built on a data cohort where all patients received thrombolysis therapy with standard dosage, ignoring the impact of treatment options on the outcome. Only five of the previous studies took treatment options into consideration: they achieved the patient selection by introducing the treatment option into the input features (thrombolysis or placebo, using standard or low dosage): by predicting favorable/non-favorable outcome for each patient, the machine learning model could give insights into what is expected to the patient under certain treatment option, as a result helping a clinician to decide the safety and efficiency of thrombolysis therapy for the patient: Kent et al. (34, 36), Sung et al. (25), and Tang et al. (40) developed a model to predict expected outcome for patients with placebo treatment vs. thrombolysis treatment. A study from Taiwan in 2020 (30) forecasted the poststroke SICH and 3-month mortality for patients receiving standard thrombolytic dosage vs. lower thrombolytic dosage.

By defining the objective of the machine learning model as foreseeing what is expected of the patient under certain treatment options, previous studies considered treatment options as an input. Together with all the other clinical variables, treatment option was processed by the machine learning classification algorithm as predictors of patients' outcome. However, a treatment option in a real clinical situation is a decision made by neurologists based on the patient's clinical profile, financial condition, and a clear understanding of the current evidence (44). Therefore, treatment options should be statistically correlated with all the other clinical variables in the input data, which will influence the prediction and inference ability of machine learning models. On the one hand, machine learning models become unstable in the presence of high feature correlations (45): for linear models, multicollinearity can yield solutions numerically unstable; for tree-based models which are good at detecting interactions between different features, highly correlated features can mask these interactions. Besides, high correlation can lead to unreliable inference conclusions. For example, the result of the study from Taiwan in 2020 (30) showed a high correlation between aging and a lower dosage of thrombolysis. Meanwhile, the model also inferred that patients who received a lower dosage had a higher mortality rate in a 3-month follow-up. The inference conclusion is not reliable due to the unclear cause of the higher mortality rate during 3-month follow-up: given the high correlation between two input variables: the aging and the lower dosage, we are not certain whether the age or the lower dosage results in the higher mortality rate. We propose that before constructing the outcome prediction model, an inference machine learning model to statistically test if the treatment option is correlated with certain clinical features is necessary. If a high correlation is found, the treatment option should be excluded from the input variable set and the inference model could also help to summarize the treatment option making experience from the large dataset and infer the important clinical factors to be considered when deciding thrombolysis eligibility and dosage. If a high correlation is not found in the dataset, the treatment option could be maintained as an input.

Another point to be noted is that previous studies did not include thrombectomy following thrombolysis as a treatment option. Since 2015, randomized clinical trials have demonstrated that mechanical thrombectomy improves functional outcomes in patients with stroke over intravenous thrombolysis alone (46). The latest European thrombolysis guidelines published in 2021 also suggested that further clinical trials are needed to inform clinical decision-making with regard to the use of thrombolysis before thrombectomy in patients with large vessel occlusion (47). The emergence of mechanical thrombectomy raises interest in thrombolytic strategies for ischemic stroke in the thrombectomy era. To be eligible in real clinical situations in the future, a machine learning based thrombolysis therapy decision support tool needs to stay tuned to this thrombectomy trend.

## 5. Clinical feature selection

Selecting significant input variables, in other words, feature selection is an important prerequisite for machine learning model construction. Feature selection is the process of choosing an optimal subset of features that best captures the human experience required to achieve the clinical goal of the machine learning model, among all the available features in the patient's clinical profile. Feature selection serves to decrease the number of input variables to both reduce the computational cost of modeling and avoid overfitting. Previous studies performed feature selection with a combination of clinical and statistical judgment: initially selected clinical features were identified by neurologists with clinical expertise or based on related studies, feature engineering was then adopted by some studies to transform raw data (we will explore feature engineering in details in the Section 6); stepwise model building (19, 25, 27, 29, 34, 39), univariate analysis (17, 20, 28, 30, 33, 38, 43, 48), multivariable analysis using logistic regression (16, 21, 24, 26, 31, 32), plots displaying the pattern of predictors, and outcome (21), and Least Absolute Shrinkage and Selection Operator (LASSO) (25, 40), was performed to further select statistically significant features among initially selected features and new features generated in feature engineering.

**Figure 3:**
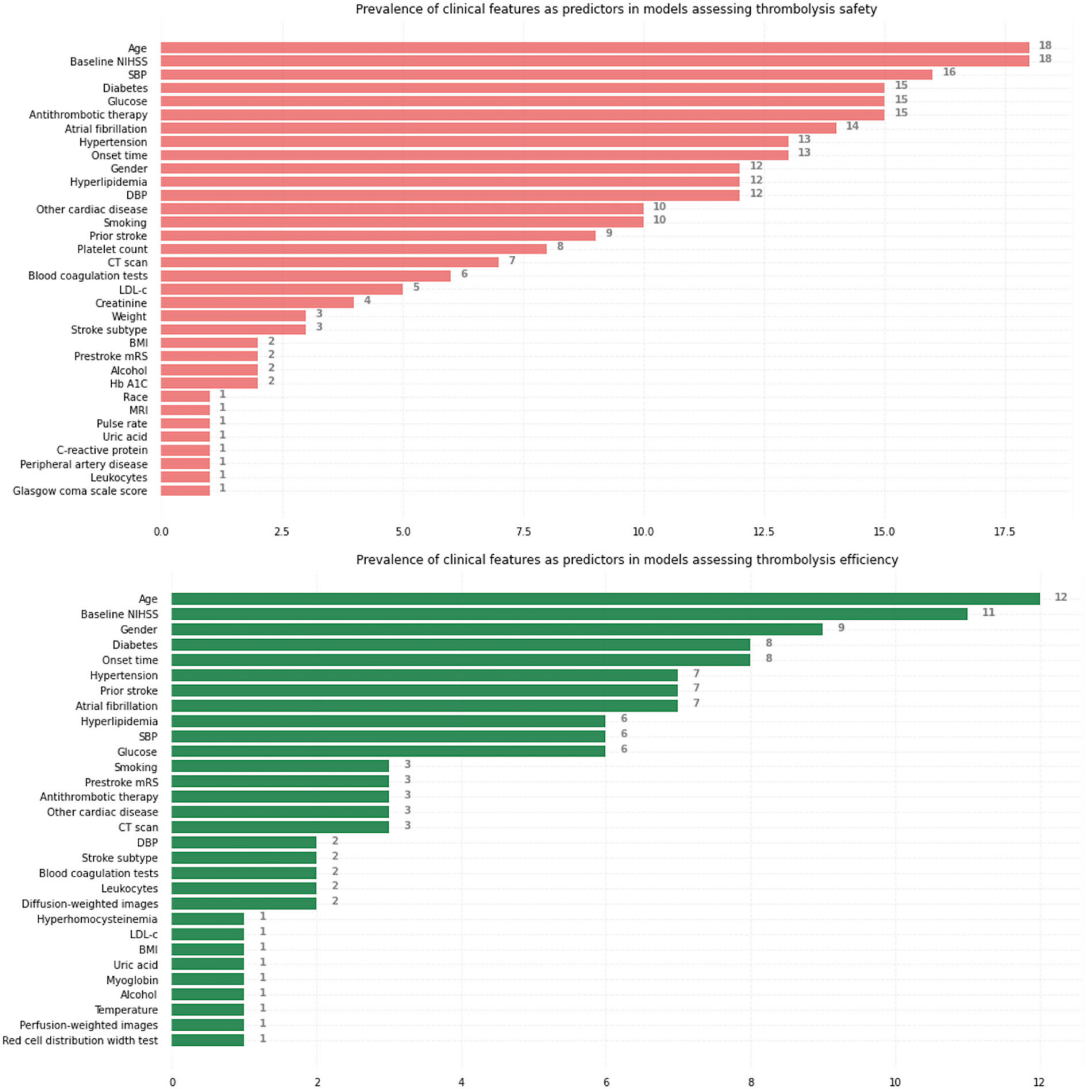
Prevalence of initially selected clinical features identified by neurologists with clinical expertise or based on related studies as predictors, respectively, in models assessing thrombolysis safety and thrombolysis efficiency. NIHSS, National Institutes of Health Stroke Scale; SBP, systolic blood pressure; DBP, diastolic blood pressure; CT, computed tomography; LDL-c, Low-density-lipoprotein cholesterol; BMI, body mass index; Hb A1C, hemoglobin A1c; MRI, magnetic resonance imaging; mRS, modified Rankin Scale.

Figure 3 summarizes the prevalence of initially selected clinical features identified by neurologists with clinical expertise or based on related studies, respectively, in models assessing thrombolysis safety and thrombolysis efficiency. Age, Baseline NIHSS, Systolic blood pressure (SBP), Diabetes, and Glucose were the five most commonly used predictors to predict safety while Age, Baseline NIHSS, Gender, Diabetes, and Onset time were the five most commonly used predictors to predict efficiency.

We noticed that only a moderate number (8 in models assessing safety and 5 in models assessing efficiency) of studies included radiological features [computerized tomography (CT) scan, magnetic resonance imaging (MRI) sequences] as model predictors. The lack of inclusion of radiological features might lead to a risk of model overfitting due to the valuable information radiological features provide regarding thrombolysis safety and efficiency (49, 50). For example, the research published in 2020 (30) developed a machine learning model predicting SICH and mortality at 3 months without any medical image based information. The cohort used to train the deep learning neural network model consists of 331 patients, a moderate sample size given the relatively large number of parameters in the neural network, while the model predicts the outcomes with a high Area under the Receiver Operating Characteristic curves (AUC) of 0.974. Given the massive information, the medical images contain regarding the thrombolysis outcome prediction (51), a model without any medical image input will normally fail to predict outcome accurately due to an incomplete patient's clinical profile and the high performance of the model in this research might be due to overfitting.

## 6. Feature engineering

In some cases, if raw clinical features are not able to capture the semantics that the human brain understands from the dataset, such as some radiological features representing penumbra and proximal/distal arterial occlusion information, feature engineering is required to create/extract features using domain knowledge.

Bentley et al. (24) leveraged a rather simple feature engineering technique for example: CT scan radiological characteristics, blood sugar, age, and baseline NIHSS are both important factors to predict the risk of SICH after thrombolysis (52). However, these separate input variables might not be able to capture the way we humans understand how these factors influence SICH. As a result, Bentley et al. (24) included a new variable SEDAN score synthesized by all the independent variables above. SEDAN score is a prediction rule for assessment of the risk of SICH (53) and can be considered as a result of feature engineering on CT scan radiological characteristics, blood sugar, age, and baseline NIHSS.

When we reviewed past related studies, we found that most of the previous models did not pay much attention to feature engineering. There is either no feature engineering (16, 22, 23, 26, 29, 30, 32, 37–39, 41–43, 48), or simple feature engineering by calculating clinical assessment scores based on past studies (18, 24, 31, 34), creating interaction terms (21, 25, 34, 36), creating dummy variables using different cutoff points (17, 19, 20, 33, 35), visual detection of radiological features (17–20, 24, 27, 28, 35). Tang et al. (40) performed an advanced radiological feature engineering by first dividing the brain into six gray matter regions plus a white matter area and then calculating the mismatch ratio between diffusion lesion and perfusion lesion in each of these seven brain areas. The newly generated mismatch features based on diffusion-weighted imaging (DWI) and perfusion-weighted imaging (PWI) represent penumbra information. We suggest that the division of the brain, especially the white matter area, could be more detailed given the fact that the infarct topography in different white matter regions could have significantly different influences on the outcome (54).

The fast progression of computer vision in recent years allows computers to better understand medical images and sometimes to extract radiological features that humans cannot see. There have already been large quantities of studies investigating the relationship between traditional clinical data and thrombolysis outcomes. Because of computer technology limitations, in the past, we could not extract advanced radiological features from medical images and there are limited studies in this field. Advanced radiological features which cannot be easily identified by human eyes can possibly offer critical information related to penumbra and, therefore, contribute immensely to early thrombolytic strategies (55). Further efforts need to be made to perform feature engineering on medical images by applying computer vision techniques to extract advanced radiological features.

We would like to propose a new penumbra related radiological feature based on a modified clinical-diffusion mismatch principle. The conventional clinical-diffusion mismatch (CDM) has been proposed as an easier alternative to the perfusion-diffusion mismatch (PDM) for selecting patients with salvageable ischemic tissue (56). It is based on the assumption that patients with severe clinical deficits, but with relatively small lesion volumes on DWI, are likely to have an ischemic penumbra (57). However, besides the infarct volume, the infarct topography can also influence the initial ischemic stroke severity dramatically. For example, according to the research by Ona WU in 2015 (54), injury to certain important functional areas, in particular motor pathways and white matter tracts, insula and putamen are associated with more severe initial symptoms and higher baseline NIHSS scores. If the lesion occupies these important functional areas, the patient can still present a rather high baseline NIHSS score without a large infarct core or a penumbra. Therefore, we propose a modified clinical-diffusion mismatch approach to better assess the penumbra: our solution will first quantify the infarct core volume in each brain functional and structural region based on Harvard-Oxford cortical and subcortical structural atlases and JHU DTI-based white-matter atlases from FSL software (58), then learn the weight of DWI infarct core volume of each brain region in the mismatch model through the machine learning algorithm that we designed. Furthermore, we propose that quantification of infarct core volume in each vascular territory could also be included as a radiological feature. Previous studies have demonstrated that if the DWI infarct lesion is found in a wide range in one large artery territory, it is very likely that the thrombus evoking the stroke is located in the large artery and endovascular treatment instead of thrombolysis is highly recommended since rtPA can hardly resolve a large thrombus (47). This vascular territory related radiological feature might contain critical information for outcome prediction for patients undergoing thrombolysis before thrombectomy.

In recent years, an increasing number of studies have investigated the impact of clot composition on the efficiency of thrombolysis.The clot/thrombus are highly heterogenous and vary in composition and organization. Fibrin-rich clots might have increased stiffness and decreased deformability compared with red blood cell-rich clots, therefore, correlating to less favorable clinical outcomes (59). The composition of a clot depends on multiple factors, including but not limited to time (60), primary sites of clot formation (61), and level of plasma Von Willebrand factor (VWF) (62). Currently, imaging evidence of clot characteristics was limited, including hyperdense middle cerebral artery sign on CT and blooming artifact on susceptibility weighted imaging (SWI) (60). The interaction term between biomarkers, clot characteristics, and imaging manifestation could be generated. We believe machine learning has the potential in inferring more hidden associations and interactions between these clot composition related features, thus providing new insights into the management of thrombolytic treatment.

**Figure 4:**
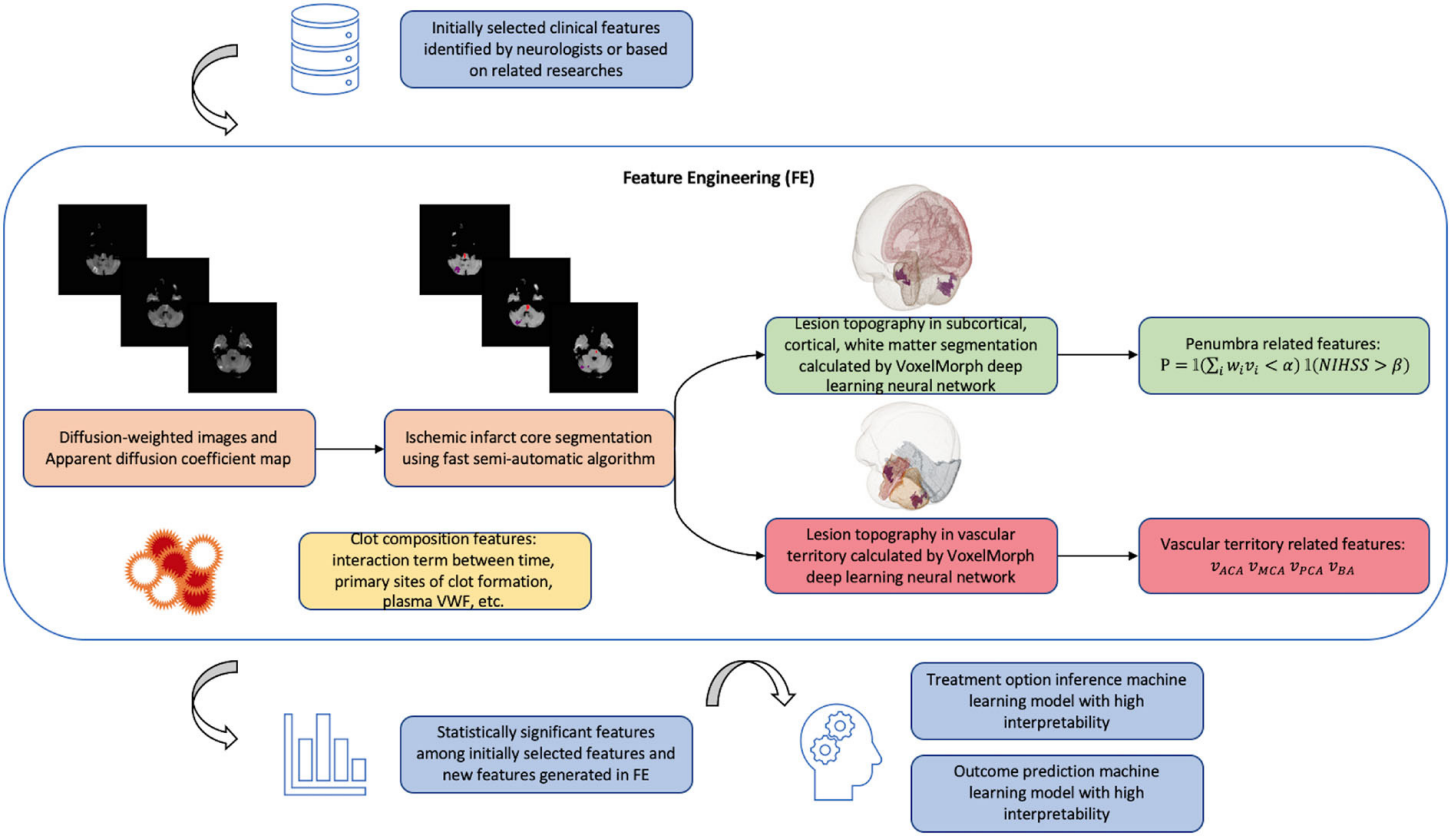
Schema of new model algorithm based on the improvements that we proposed summarized in Table 1. The fast pipeline to calculate advanced radiological features in FE was published in our previous research (68). Penumbra related features are calculated based on clinical-diffusion mismatch: $P=1\left(\underset{i}{\sum }w_{i}v_{i}<\alpha \right)1\left(NIHSS>\beta \right)$ where *i* is the index of brain region affected by ischemic lesion; *v*_*i*_ is the lesion volume in region *i*; *w*_*i*_ is the contribution of region *i* to the initial ischemic stroke severity provided by previous studies (54); $\underset{i}{\sum }w_{i}v_{i}$ is a weighted lesion volume corrected by anatomic correlates of admission stroke severity: in cases when lesion occupies important functional area such as Internal Capsule, *w*_*i*_ will increase the value of weighted lesion volume and avoid the false clinical-diffusion mismatch. *NIHSS* is the initial ischemic stroke severity score; α and β need to be tuned during machine learning training process. *P* is the product of two indicator functions. Vascular territory related features are lesion volume, respectively, in anterior cerebral artery (ACA) territory, middle cerebral artery (MCA) territory, posterior cerebral artery (PCA) territory, and basilar artery (BA) territory.

A detailed illustration of our proposed feature engineering can be found in Figure 4.

## 7. Model algorithm development

Model algorithm development is a process where we leverage computer science and statistics to design an algorithm that is able to achieve the predefined clinical goal using a training dataset. Of the many algorithms used by previous thrombolysis outcome prediction studies, some are more flexible, others are more restrictive. The more estimated parameters the model algorithm depends on, the more flexible the model is considered to be. The algorithms used by previous studies, from the most restrictive to the most flexible, were risk score (16, 17, 19–23, 33, 35), nomogram (27, 31, 32, 37–39), logistic regression (25, 26, 28, 29, 34, 36, 40, 43, 48), tree-based machine learning models (18, 29, 43, 48), support-vector machine (SVM) (18, 24, 29), and deep learning neural network (29, 30, 41, 42, 48).

In fact, when developing a machine learning algorithm, there is usually a trade-off between flexibility and interpretability (63): Inflexible algorithms have a restrictive ability to estimate the boundaries between different outcome classes, therefore, producing the predicted outcome with lower accuracy. But Inflexible algorithms are often easy to be interpreted. On the other hand, flexible algorithms generate more accurate predicted outcomes but suffer from low interpretability. Most of the previous models have a preference for restrictive models (risk score, nomogram, logistic regression, and tree-based machine learning models) for high interpretability. Regarding the flexible algorithms, there are two common ways to increase the interpretability: (1) A reactive approach to calculate individual predictor importance using the SHapley Additive exPlanations (SHAP) framework proposed by Lundberg and Lee (64). (2) A proactive approach to increasing model prediction accuracy by boosting interpretability, where a very popular example is the attention mechanism introduced in 2014 (65) to allow the deep learning neural network decoder to leverage the most relevant parts of the input vectors in a flexible manner. The latter approach is recommended however requires efforts in developing new algorithms. In order to further improve the performance of models, future studies could stay tuned to the new findings in the machine learning field and try to develop new algorithms which maintain interpretability while improving prediction accuracy compared with current machine learning algorithms.

## 8. Processing time consideration

The processing time of a thrombolytic clinical decision support tool covers three parts: time for automatic data preparation, time for automatic feature engineering, and time for machine learning algorithm running. Automatic data preparation is quite mature nowadays in the industry with the emergence of Data Engineering and can be easily and fast done through well-written Structured Query Language (SQL) script and Big Data frameworks such as MapReduce (66). A machine learning algorithm requires a long time to be trained in Developing Pipeline if the dataset is big. However, obtaining an outcome using a well-trained algorithm usually requires seconds. More attention needs to be paid to the processing time of feature engineering to extract advanced radiological features from medical images.

As we reviewed past studies, we found that only nine past studies mentioned the processing time consideration, (16, 19, 20, 22, 33–37): they chose clinical input easy to obtain in the emergency situation. However, neither of these nine studies included advanced radiological features from medical images due to the difficulty to calculate these features in emergencies.

We noticed that in previous studies, the radiological features, such as penumbra and infarct core volume from DWI and PWI (40), ASPECT scores from CT (18), and SICH-prognostic SEDAN/HAT scores (24), were extracted manually using traditional pipeline in open-source software. Using the traditional pipeline to interpret medical images is accurate but slow. Recent computer vision studies designed some deep learning based pipelines to automatically interpret medical images. These deep learning pipelines are able to achieve an acceptable similarity with the traditional pipeline while greatly shortening the processing time (67). Regarding the feature engineering that we proposed in Figure 4, we have also developed deep learning based fast-processing pipeline to calculate the lesion volume in each brain structural region and vascular territory (68). Our study has been published in the Proceedings of the 2021 IEEE International Conference on Bioinformatics and Biomedicine. Our pipeline takes diffusion sequences of raw MRI images in Digital imaging and communications in medicine (DICOM) format as input: DWI and its associated apparent diffusion coefficient (ADC), and can calculate the lesion volume in each brain structural region and vascular territory much faster than baseline pipeline in average 138 s on a normal PC CPU with processor 2.6 GHz Intel Core i5 and memory 8 Go 1,600 MHz DDR3. In terms of dice score, our study is able to achieve on average 80.3% similarity with the baseline pipeline. In the future, more efforts could be made to shorten the radiological pipeline processing time.

## 9. Discussion and conclusion

Previous personalized predictive models employed in the decision-making of thrombolysis basically stay in the research stage and have a long way to go before being applied in real clinical practice. In Table 1, we made a summary of previous studies in terms of the five criteria a machine learning model should meet in order to be accurate and feasible in assisting thrombolysis therapy (Figure 2), as well as the corresponding proposed improvements. In Figure 4, we also provide a schema illustrating the new model algorithm based on the improvements we proposed.

**Table 1:** Summary of previous studies in terms of the five criteria a machine learning model should meet in order to be accurate and feasible in assisting thrombolysis therapy (Figure 2), as well as the corresponding proposed improvements.

	**Reasonable clinical goal definition**	**Optimal clinical input selection**	**Feature engineering**	**Model with high interpretability**	**Model with short processing time**
Previous researches	(1) Only 2 studies assessed both efficiency and safety; (2) The clinical outputs of models predicting safety are all poststroke symptomatic intracerebral hemorrhage while the clinical outputs of models predicting efficiency vary; (3) Only five of previous researches took treatment option into consideration by introducing the treatment option into the input features (thrombolysis or placebo, using standard or low dosage)	Feature selection was performed with a combination of clinical and statistical judgement, only a moderate number (eight in models assessing safety and five in models assessing efficiency) of researches included radiological features (CT scan, MRI sequences) as model predictors	Most of previous models did not pay much attention to feature engineering. There is either no feature engineering, or simple feature engineering by calculating clinical assessment score, creating interaction terms, creating dummy variables, visual detection of radiological features	Most of previous models have a preference for restrictive models (risk score, nomogram, logistic regression and tree-based machine learning models) for the high interpretability	Only nine past studies mentioned the processing time consideration: they chose clinical input easy to obtain in the emergency situation. Neither of these nine studies included advanced radiological features from medical images due to the difficulty to calculate these features in emergency
Proposed improvements	(1) A model assisting in thrombolysis therapy needs to assess both efficiency and safety; (2) To provide comprehensive thrombolysis efficiency assessment, both early and long-term outcome prediction are required; (3) Before constructing the outcome prediction model, an inference machine learning model to statistically test if treatment option is correlated with certain clinical features is necessary, thrombectomy following thrombolysis needs to be considered as a treatment option as well	Inclusion of radiological features are needed. The lack of inclusion of radiological features might lead to a risk of model overfitting due to the valuable information radiological features provide regarding to thrombolysis safety and efficiency	Advanced radiologial features representing penumbra and proximal/distal arterial occlusion information could be computed using computer vision. Interaction term between biomarkers, clot characteristics, and imaging manifestation could be generated to represent clot composition	Flexible algorithms have higher accuracy. A proactive approach could be adopted to increase flexible model prediction accuracy by boosting interpretability	Deep learning based pipelines could be used to automatically interpret medical images to obtain advanced radiological features in a short time

The accuracy of a machine learning model largely depends on appropriate model conceptualization, requiring a reasonable definition of the clinical goal, clinical input, and feature engineering based on comprehensive neurological domain knowledge summarized from past clinical trials. Efficiency and safety assessment are both required to better select patients who could benefit the most from thrombolysis. Poststroke symptomatic intracerebral hemorrhage is an appropriate indicator for thrombolysis safety. To provide a comprehensive thrombolysis efficiency assessment, both early and long-term outcome predictions are required. Given the possible high correlation between treatment option and clinical profile, an inference machine learning model to statistically test if treatment option is correlated with certain clinical features is necessary before constructing the outcome prediction model. The possible treatment options are placebo, thrombolysis with a low dosage, thrombolysis with standard dosage, and thrombolysis followed by thrombectomy. The lack of advanced radiological features representing penumbra and proximal/distal arterial occlusion information are commonly found in previous studies. In recent years, with an increasing number of studies investigating the impact of clot composition on the efficiency of thrombolysis, the interaction term between biomarkers, clot characteristics, and imaging manifestation could be generated to represent clot composition.

The feasibility of a machine learning model, on the other hand, needs to be achieved by elaborate computer science algorithms to increase the interpretability of flexible algorithms and shorten the processing time of the pipeline interpreting medical images. Previous models tend to adopt a passive way in terms of feasibility: they chose restrictive models with low accuracy for high interpretability and avoided advanced radiological features due to the difficulty to calculate them in an emergency. Recent advancements in computer science would allow future models to achieve feasibility while not compromising accuracy.

In summary, an accurate and feasible machine learning model in assisting thrombolysis therapy should be both clinical-evidence orientated and algorithm orientated, thus requiring interdisciplinary collaboration between neurologists, who could provide comprehensive domain knowledge, and computer scientists, who could improve the performance of current algorithms.

## Author contributions

HS and HD reviewed previous studies and drafted the manuscript. XC, QM, ZS, and WC helped draft the manuscript. All authors read and approved the submitted version.

## Conflict of interest

The authors declare that the research was conducted in the absence of any commercial or financial relationships that could be construed as a potential conflict of interest.

## Publisher's note

All claims expressed in this article are solely those of the authors and do not necessarily represent those of their affiliated organizations, or those of the publisher, the editors and the reviewers. Any product that may be evaluated in this article, or claim that may be made by its manufacturer, is not guaranteed or endorsed by the publisher.

## References

[1] Feigin VL Norrving B Mensah GA. Global burden of stroke. Circul Res. (2017) 120:439–48. 10.1161/CIRCRESAHA.116.308413 28154096

[2] Miller EL Murray L Richards L Zorowitz RD Bakas T Clark P. Comprehensive overview of nursing and interdisciplinary rehabilitation care of the stroke patient. Stroke. (2010) 41:2402–48. 10.1161/STR.0b013e3181e7512b 20813995

[3] Lloyd-Jones D Adams R Carnethon M Simone GD Ferguson TB Flegal K. Heart disease and stroke statistics-2009 update. Circulation. (2009) 119:480–6. 10.1161/CIRCULATIONAHA.108.191259 19171871

[4] Adams HP Bendixen BH Kappelle LJ Biller J Love BB Gordon DL. Classification of subtype of acute ischemic stroke. Definitions for use in a multicenter clinical trial. TOAST. Trial of Org 10172 in Acute Stroke Treatment. Stroke. (1993) 24:35–41. 10.1161/01.STR.24.1.35 7678184

[5] Broderick JP. Recanalization therapies for acute ischemic stroke. Semin Neurol. (1998) 18:471–84. 10.1055/s-2008-1040900 9932618

[6] Jivan K Ranchod K Modi G. Management of ischaemic stroke in the acute setting: review of the current status : review article. Cardiovasc J Afr. (2013) 24:86–92. 10.5830/CVJA-2013-001 23736133 PMC3721925

[7] Wardlaw JM Murray V Berge E del Zoppo GJ. Thrombolysis for acute ischaemic stroke. Cochrane Database Syst Rev. (2014) 2014:CD000213. 10.1002/14651858.CD000213.pub3 25072528 PMC4153726

[8] Miller DJ Simpson JR Silver B. Safety of thrombolysis in acute ischemic stroke: a review of complications, risk factors, and newer technologies. Neurohospitalist. (2011) 1:138–47. 10.1177/1941875211408731 23983849 PMC3726129

[9] Dávalos A. Thrombolysis in acute ischemic stroke: successes, failures, and new hopes. Cerebrovasc Dis. (2005) 20:135–9. 10.1159/000089367 16327264

[10] Stinear CM Smith MC Byblow WD. Prediction tools for stroke rehabilitation. Stroke. (2019) 50:3314–22. 10.1161/STROKEAHA.119.025696 31610763

[11] Echouffo-Tcheugui JB Woodward M Kengne AP. Predicting a post-thrombolysis intracerebral hemorrhage: a systematic review. J Thromb Haemost. (2013) 11:862–71. 10.1111/jth.12186 23469771

[12] Anderson CS Robinson T Lindley RI Arima H Lavados PM Lee TH. Low-dose versus standard-dose intravenous alteplase for octogenerian acute ischemic stroke patients: a multicenter prospective cohort study. J Neurol Sci. (2019) 399:76–81. 10.1056/NEJMoa1515510 30780072

[13] Lyden PD. Thrombolytic therapy for acute ischemic stroke. Stroke. (2019) 50:2597–603. 10.1161/STROKEAHA.119.025699 31327316 PMC6710099

[14] Mitchell TM. Machine Learning. McGraw-Hill (1997). Available online at: https://books.google.com.hk/books?id=EoYBngEACAAJ

[15] Man S Xian Y Holmes DN Matsouaka RA Saver JL Smith EE. Association between thrombolytic door-to-needle time and 1-year mortality and readmission in patients with acute ischemic stroke. JAMA. (2020) 323:2170–84. 10.1001/jama.2020.5697 32484532 PMC7267850

[16] Cucchiara B Tanne D Levine SR Demchuk AM Kasner S. A risk score to predict intracranial hemorrhage after recombinant tissue plasminogen activator for acute ischemic stroke. J Stroke Cerebrovasc Dis. (2008) 17:331–3. 10.1016/j.jstrokecerebrovasdis.2008.03.012 18984422

[17] Lou M Safdar A Mehdiratta M Kumar S Schlaug G Caplan L. The HAT score. Neurology. (2008) 71:1417–23. 10.1212/01.wnl.0000330297.58334.dd 18955684 PMC2676961

[18] Dharmasaroja P Dharmasaroja PA. Prediction of intracerebral hemorrhage following thrombolytic therapy for acute ischemic stroke using multiple artificial neural networks. Neurol Res. (2012) 34:120–8. 10.1179/1743132811Y.0000000067 22333462

[19] Strbian D Engelter S Michel P Meretoja A Sekoranja L Ahlhelm FJ. Symptomatic intracranial hemorrhage after stroke thrombolysis: the SEDAN Score. Ann Neurol. (2012) 71:634–41. 10.1002/ana.23546 22522478

[20] Mazya M Egido JA Ford GA Lees KR Mikulik R Toni D. Predicting the risk of symptomatic intracerebral hemorrhage in ischemic stroke treated with intravenous alteplase. Stroke. (2012) 43:1524–31. 10.1161/STROKEAHA.111.644815 22442178

[21] Menon BK Saver JL Prabhakaran S Reeves M Liang L Olson DM. Risk score for intracranial hemorrhage in patients with acute ischemic stroke treated with intravenous tissue-type plasminogen activator. Stroke. (2012) 43:2293–9. 10.1161/STROKEAHA.112.660415 22811458

[22] Saposnik G Guzik AK Reeves M Ovbiagele B Johnston SC. Stroke prognostication using age and NIH stroke scale. Neurology. (2013) 80:21–8. 10.1212/WNL.0b013e31827b1ace 23175723 PMC3589202

[23] Flint AC Gupta R Smith WS Kamel H Faigeles BS Cullen SP. The THRIVE score predicts symptomatic intracerebral hemorrhage after intravenous tPA administration in SITS-MOST. Int J Stroke. (2014) 9:705–10. 10.1111/ijs.12335 25042855

[24] Bentley P Ganesalingam J Carlton Jones AL Mahady K Epton S Rinne P. Prediction of stroke thrombolysis outcome using CT brain machine learning. NeuroImage. (2014) 4:635–40. 10.1016/j.nicl.2014.02.003 24936414 PMC4053635

[25] Sung LJ Kyung KC Jihoon K Jong-Moo P Hwan PT Bok LK. A novel computerized clinical decision support system for treating thrombolysis in patients with acute ischemic stroke. J Stroke. (2015) 17:199-209. 10.5853/jos.2015.17.2.199 26060807 PMC4460339

[26] Lokeskrawee T Muengtaweepongsa S Patumanond J Tiamkao S Thamangraksat T Phankhian P. Prediction of symptomatic intracranial hemorrhage after intravenous thrombolysis in acute ischemic stroke: the symptomatic intracranial hemorrhage score. J Stroke Cerebrovasc Dis. (2017) 26:2622–9. 10.1016/j.jstrokecerebrovasdis.2017.06.030 28826584

[27] Cappellari M Turcato G Forlivesi S Zivelonghi C Bovi P Bonetti B. STARTING-SICH nomogram to predict symptomatic intracerebral hemorrhage after intravenous thrombolysis for stroke. Stroke. (2018) 49:397–404. 10.1161/STROKEAHA.117.018427 29311264

[28] Nisar T Hanumanthu R Khandelwal P. Symptomatic intracerebral hemorrhage after intravenous thrombolysis: predictive factors and validation of prediction models. J Stroke Cerebrovasc Dis. (2019) 28:104360. 10.1016/j.jstrokecerebrovasdis.2019.104360 31501036

[29] Wang F Huang Y Xia Y Zhang W Fang K Zhou X. Personalized risk prediction of symptomatic intracerebral hemorrhage after stroke thrombolysis using a machine-learning model. Therap Adv Neurol Disord. (2020) 13:1756286420902358. 10.1177/1756286420902358 35173804 PMC8842114

[30] Chung CC Chan L Bamodu OA Hong CT Chiu HW. Artificial neural network based prediction of postthrombolysis intracerebral hemorrhage and death. Sci Rep. (2020) 10:20501. 10.1038/s41598-020-77546-5 33239681 PMC7689530

[31] Wu Y Chen H Liu X Cai X Kong Y Wang H. A new nomogram for individualized prediction of the probability of hemorrhagic transformation after intravenous thrombolysis for ischemic stroke patients. BMC Neurol. (2020) 20:426. 10.1186/s12883-020-02002-w 33234113 PMC7685652

[32] Zhou Z Yin X Niu Q Liang S Mu C Zhang Y. Risk factors and a nomogram for predicting intracranial hemorrhage in stroke patients undergoing thrombolysis. Neuropsychiatr Dis Treat. (2020) 2020:16:1189–97. 10.2147/NDT.S250648 32494138 PMC7231854

[33] Soni M Wijeratne T Ackland DC. A risk score for prediction of symptomatic intracerebral haemorrhage following thrombolysis. Int J Med Inform. (2021) 156:104586. 10.1016/j.ijmedinf.2021.104586 34649112

[34] Kent DM Selker HP Ruthazer R Bluhmki E Hacke W. The stroke (2013) thrombolytic predictive instrument. Stroke. (2006) 37:2957–62. 10.1161/01.STR.0000249054.96644.c6 17068305

[35] Strbian D Meretoja A Ahlhelm FJ Pitkäniemi J Lyrer P Kaste M. Predicting outcome of IV thrombolysis-treated ischemic stroke patients. Neurology. (2012) 78:427–32. 10.1212/WNL.0b013e318245d2a9 22311929

[36] Kent DM Ruthazer R Decker C Jones PG Saver JL Bluhmki E. Development and validation of a simplified Stroke-Thrombolytic Predictive Instrument. Neurology. (2015) 85:942–9. 10.1212/WNL.0000000000001925 26291280 PMC4567461

[37] Cappellari M Turcato G Forlivesi S Bagante F Cervellin G Lippi G. The START nomogram for individualized prediction of the probability of unfavorable outcome after intravenous thrombolysis for stroke. Int J Stroke. (2018) 13:700–6. 10.1177/1747493018765490 29540109

[38] Lv S Song Y Zhang FL Yan XL Chen J Gao L. Early prediction of the 3-month outcome for individual acute ischemic stroke patients who received intravenous thrombolysis using the N2H3 nomogram model. Therap Adv Neurol Disord. (2020) 13:1756286420953054. 10.1177/1756286420953054 35173805 PMC8842152

[39] Huang L Li F Huang C Luo Y Liu G. A novel nomogram for predicting poor 6-month function in patients with acute ischemic stroke receiving thrombolysis. J Cardiovasc Nurs. (2021). 10.1097/JCN.0000000000000843. [Epub ahead of print]. 34321431

[40] Tang TY Jiao Y Cui Y Zeng CH Zhao DL Zhang Y. Development and validation of a penumbra-based predictive model for thrombolysis outcome in acute ischemic stroke patients. EBioMedicine. (2018) 35:251–9. 10.1016/j.ebiom.2018.07.028 30146341 PMC6154778

[41] Bacchi S Zerner T Oakden-Rayner L Kleinig T Patel S Jannes J. Deep learning in the prediction of ischaemic stroke thrombolysis functional outcomes: a pilot study. Acad Radiol. (2020) 27:e19–23. 10.1016/j.acra.2019.03.015 31053480

[42] Chen Z Li Q Li R Zhao H Li Z Zhou Y. Ensemble learning accurately predicts the potential benefits of thrombolytic therapy in acute ischemic stroke. Quant Imaging Med Surg. (2021) 11:3978–89. 10.21037/qims-21-33 34476183 PMC8339640

[43] Zhu B Zhao J Cao M Du W Yang L Su M. Predicting 1-hour thrombolysis effect of r-tPA in Patients with acute ischemic stroke using machine learning algorithm. Front Pharmacol. (2022) 12:759782. 10.3389/fphar.2021.759782 35046804 PMC8762247

[44] Dong Y Cao W Cheng X Fang K Wu F Yang L. Low-dose intravenous tissue plasminogen activator for acute ischaemic stroke: an alternative or a new standard? Stroke Vasc Neurol. (2016) 1:115–21. 10.1136/svn-2016-000033 28959472 PMC5435201

[45] Toloi L Lengauer T. Classification with correlated features: unreliability of feature ranking and solutions. Bioinformatics. (2011) 27:1986–94. 10.1093/bioinformatics/btr300 21576180

[46] Gauberti M Martinez de Lizarrondo S Vivien D. Thrombolytic strategies for ischemic stroke in the thrombectomy era. J Thromb Haemost. (2021) 19:1618–28. 10.1111/jth.15336 33834615

[47] Berge E Whiteley W Audebert H Marchis GD Fonseca AC Padiglioni C. European Stroke Organisation (ESO) guidelines on intravenous thrombolysis for acute ischaemic stroke. Eur Stroke J. (2021) 6:I–LXII. 10.1177/2396987321989865 33817340 PMC7995316

[48] Chung CC Bamodu OA Hong CT Chan L Chiu HW. Application of machine learning-based models to boost the predictive power of the SPAN index. Int J Neurosci. (2021) 1–11. 10.1080/00207454.2021.1881092 33499706

[49] Gravanis I Tsirka SE. Tissue-type plasminogen activator as a therapeutic target in stroke. Expert Opin Therap Targets. (2008) 12:159–70. 10.1517/14728222.12.2.159 18208365 PMC3824365

[50] Zou M Churilov L He A Campbell B Davis SM Yan B. Hyperdense middle cerebral artery sign is associated with increased risk of hemorrhagic transformation after intravenous thrombolysis for patients with acute ischaemic stroke. J Clin Neurosci. (2013) 20:984–7. 10.1016/j.jocn.2012.10.013 23664409

[51] El-Koussy M Schroth G Brekenfeld C Arnold M. Imaging of acute ischemic stroke. Eur Neurol. (2014) 72:309–16. 10.1159/000362719 25323674

[52] Muengtaweepongsa S Prapa-Anantachai P Dharmasaroja PA Rukkul P Yodvisitsak P. External validation of the SEDAN score: the real world practice of a single center. Ann Indian Acad Neurol. (2015) 18:181–6. 10.4103/0972-2327.150592 26019416 PMC4445194

[53] Mazya MV Bovi P Castillo J Jatuzis D Kobayashi A Wahlgren N. External validation of the SEDAN score for prediction of intracerebral hemorrhage in stroke thrombolysis. Stroke. (2013) 44:1595–600. 10.1161/STROKEAHA.113.000794 23632975

[54] Wu O Cloonan L Mocking SJT Bouts MJRJ Copen WA Cougo-Pinto PT. Role of acute lesion topography in initial ischemic stroke severity and long-term functional outcomes. Stroke. (2015) 46:2438–44. 10.1161/STROKEAHA.115.009643 26199314 PMC4550548

[55] Bhaskar S Stanwell P Cordato D Attia J Levi C. Reperfusion therapy in acute ischemic stroke: dawn of a new era? BMC Neurol. (2018) 18:8. 10.1186/s12883-017-1007-y 29338750 PMC5771207

[56] Davalos A Blanco M Pedraza S Leira R Castellanos M Pumar JM. The clinical-DWI mismatch. Neurology. (2004) 62:2187–92. 10.1212/01.WNL.0000130570.41127.EA 15210880

[57] Lansberg MG Thijs VN Hamilton S Schlaug G Bammer R Kemp S. Evaluation of the clinical-diffusion and perfusion-diffusion mismatch models in DEFUSE. Stroke. (2007) 38:1826–30. 10.1161/STROKEAHA.106.480145 17495217 PMC3985733

[58] Jenkinson M Beckmann CF Behrens TEJ Woolrich MW Smith SM. FSL. NeuroImage. (2012) 62:782–90. 10.1016/j.neuroimage.2011.09.015 21979382

[59] Jolugbo P Arins RAS. Thrombus composition and efficacy of thrombolysis and thrombectomy in acute ischemic stroke. Stroke. (2021) 52:1131–42. 10.1161/STROKEAHA.120.032810 33563020 PMC7610448

[60] Czaplicki C Albadawi H Partovi S Gandhi RT Quencer K Deipolyi AR. Can thrombus age guide thrombolytic therapy? Cardiovasc Diagn Ther. (2017) 7(Suppl. 3):S186–96. 10.21037/cdt.2017.11.05 29399522 PMC5778517

[61] Sporns PB Hanning U Schwindt W Velasco A Minnerup J Zoubi T. Ischemic stroke. Stroke. (2017) 48:2206–10. 10.1161/STROKEAHA.117.016590 28626055

[62] Wieberdink RG van Schie MC Koudstaal PJ Hofman A Witteman JCM de Maat MPM. High von Willebrand factor levels increase the risk of stroke. Stroke. (2010) 41:2151–6. 10.1161/STROKEAHA.110.586289 20798373

[63] James G Witten D Hastie T Tibshirani R. An Introduction to Statistical Learning: with Applications in R. Springer (2013). Available online at: https://faculty.marshall.usc.edu/gareth-james/ISL/. 10.1007/978-1-4614-7138-7

[64] Lundberg S Lee SI. A unified approach to interpreting model predictions. In: NIPS'17: Proceedings of the 31st International Conference on Neural Information Processing Systems (Long Beach, CA). (2017).

[65] Bahdanau D Cho K Bengio Y. Neural machine translation by jointly learning to align and translate. arXiv:1409.0473. (2014). 10.48550/arXiv.1409.0473

[66] Dean J Ghemawat S. MapReduce: simplified data processing on large clusters. In: OSDI'04: Sixth Symposium on Operating System Design and Implementation. San Francisco, CA (2004). p. 137–50.

[67] Balakrishnan G Zhao A Sabuncu MR Guttag J Dalca AV. VoxelMorph: a learning framework for deformable medical image registration. IEEE Trans Med Imaging. (2019) 38:1788–800. 10.1109/TMI.2019.2897538 30716034

[68] Shao H Chan L Chen F Ma Q Shao Z Du H. A fast-processing pipeline for three-dimensional visualization of acute ischemic stroke lesion topography. In: 2021 IEEE International Conference on Bioinformatics and Biomedicine (BIBM) (Houston, TX). (2021). p. 3207–214. 10.1109/BIBM52615.2021.9669719

