# Supplementary material for: The feasibility and accuracy of machine learning in improving safety and efficiency of thrombolysis for patients with stroke: Literature review and proposed improvements
Source: Front Neurol. 2022 Oct 20;13:934929. doi: 10.3389/fneur.2022.934929 (PMC9630915; doi:10.3389/fneur.2022.934929)
Supplement: Supplementary file 1 [file Table_1.DOCX]

**Table 1.** Detailed information of the representative studies reviewed in this paper.

| No | Title | Author and year |
| --- | --- | --- |
| 1 | The Stroke–Thrombolytic Predictive Instrument: A Predictive Instrument for Intravenous Thrombolysis in Acute Ischemic Stroke | Kent et al, 2006 |
| 2 | A Risk Score to Predict Intracranial Hemorrhage After Recombinant Tissue Plasminogen Activator for Acute Ischemic Stroke | Cucchiara et al, 2008 |
| 3 | The HAT Score : A simple grading scale for predicting hemorrhage after thrombolysis | Lou et al, 2008 |
| 4 | Prediction of intracerebral hemorrhage following thrombolytic therapy for acute ischemic stroke using multiple artificial neural networks | Dharmasaroja et al, 2012 |
| 5 | Predicting outcome of IV thrombolysis–treated ischemic stroke patients The DRAGON score | Strbian et al, 2012 |
| 6 | Symptomatic Intracranial Hemorrhage after Stroke Thrombolysis: The SEDAN Score | Strbian et al, 2012 |
| 7 | Predicting the Risk of Symptomatic Intracerebral Hemorrhage in Ischemic Stroke Treated With Intravenous Alteplase Safe Implementation of Treatments in Stroke (SITS) Symptomatic Intracerebral Hemorrhage Risk Score | Mazya et al, 2012 |
| 8 | Risk Score for Intracranial Hemorrhage in Patients With Acute Ischemic Stroke Treated With Intravenous Tissue-Type Plasminogen Activator | Menon et al, 2012 |
| 9 | Stroke Prognostication using Age and NIH Stroke Scale: SPAN-100 | Saposnik et al, 2012 |
| 10 | The THRIVE score predicts symptomatic intracerebral hemorrhage after intravenous tPA administration in SITS-MOST | Flint et al, 2014 |
| 11 | Prediction of stroke thrombolysis outcome using CT brain machine learning | Bentley et al, 2014 |
| 12 | A Novel Computerized Clinical Decision Support System for Treating Thrombolysis in Patients with Acute Ischemic Stroke | Lee et al, 2015 |
| 13 | Development and validation of a simplified Stroke–Thrombolytic Predictive Instrument | Kent et al, 2015 |
| 14 | Prediction of Symptomatic Intracranial Hemorrhage after Intravenous Thrombolysis in Acute Ischemic Stroke: The Symptomatic Intracranial Hemorrhage Score | Lokeskrawee et al, 2017 |
| 15 | STARTING-SICH Nomogram to Predict Symptomatic Intracerebral Hemorrhage After Intravenous Thrombolysis for Stroke | Cappellari et al, 2018 |
| 16 | Development and validation of a penumbra-based predictive model for thrombolysis outcome in acute ischemic stroke patients | Tang et al, 2018 |
| 17 | The START nomogram for individualized prediction of the probability of unfavorable outcome after intravenous thrombolysis for stroke | Cappellar et al, 2018 |
| 18 | Deep Learning in the Prediction of Ischemic Stroke Thrombolysis Functional Outcomes: A Pilot Study | Bacchi et al, 2019 |
| 19 | Symptomatic Intracerebral Hemorrhage after Intravenous Thrombolysis: Predictive Factors and Validation of Prediction Models | Nisar et al, 2019 |
| 20 | Personalized risk prediction of symptomatic intracerebral hemorrhage after stroke thrombolysis using a machine-learning model | Wang et al, 2020 |
| 21 | Artificial neural network based prediction of postthrombolysis intracerebral hemorrhage and death | Chung et al, 2020 |
| 22 | Early prediction of the 3-month outcome for individual acute ischemic stroke patients who received intravenous thrombolysis using the N2H3 nomogram model | Lv et al, 2020 |
| 23 | A new nomogram for individualized prediction of the probability of hemorrhagic transformation after intravenous thrombolysis for ischemic stroke patients | Wu et al, 2020 |
| 24 | Risk Factors and a Nomogram for Predicting Intracranial Hemorrhage in Stroke Patients Undergoing Thrombolysis | Zhou et al, 2020 |
| 25 | Ensemble learning accurately predicts the potential benefits of thrombolytic therapy in acute ischemic stroke | Chen et al, 2021 |
| 26 | A Novel Nomogram for Predicting Poor 6-Month Function in Patients With Acute Ischemic Stroke Receiving Thrombolysis | Huang et al, 2021 |
| 27 | Application of machine learning-based models to boost the predictive power of the SPAN index | Chung et al, 2021 |
| 28 | A risk score for prediction of symptomatic intracerebral haemorrhage following thrombolysis | Soni et al, 2021 |
| 29 | Predicting 1-Hour Thrombolysis Effect of r-tPA in Patients With Acute Ischemic Stroke Using Machine Learning Algorithm | Zhu et al, 2022 |

| No | Objectives of model | Clinical output | Reperfusion treatment option | Feature selection and feature engineering strategy | Model building and interpretability | Processing time consideration |
| --- | --- | --- | --- | --- | --- | --- |
| 1 | Efficiency | Outcomes at opposite ends of the functional outcome scale (mRS $\leq1$ and mRS$\geq$5) | Thrombolysis and placebo | Only variables previously demonstrated to be prognostically important, or likely to modify treatment effect were initially selected: Age, Gender, SBP, Onset time, NIHSS, Glucose, Diabetes, Prior stroke, Hypertension, Atrial fibrillation, Other cardiac disease and CT scan.  In feature engineering, ASPECT score was computed based on CT scan; all initially selected variables were also interacted with treatment to create interaction term.  Stepwise model building was performed to further select statistically significant features among initially selected features and new features generated in feature engineering. | Regression model with high interpretability | Yes, the model is based on variables which are easily obtainable for real-time pretreatment use |
| 2 | Safety | Poststroke SICH | Thrombolysis | Only variables previously identified as factors associated with increased risk of Poststroke SICH were initially selected: Age, Diabetes, Atrial fibrillation, Other cardiac disease, Antithrombotic therapy, NIHSS, SBP, Diastolic blood pressure, Glucose, Platelet count.  No feature engineering was performed.  Multivariable analyses using logistic regression models identified statistically significant features among initially selected features. | A simple risk score with high interpretability | Yes, the model is based on variables that were easily obtainable |
| 3 | Safety | Poststroke SICH | Thrombolysis | Only variables previously identified as factors associated with increased risk of Poststroke SICH were initially selected: Age, NIHSS, Diabetes, Glucose, CT scan, Smoking, Antithrombotic therapy.  In feature engineering, visible hypodensity in MCA territory on CT was examined. Dummy variables were created based on all features using different thresholds and cutoff points based on reported values in the literature.  Prognostic ability of various combinations of dummy variables was examined, the top four dummy variables with highest odd ratio were selected. | A simple risk score with high interpretability | No |
| 4 | Safety | Poststroke SICH | Thrombolysis | Only variables previously identified as factors associated with increased risk of Poststroke SICH were initially selected: Gender, Age, Hypertension, Diabetes, Hyperlipidemia, Atrial fibrillation, Other cardiac disease, CT scan, Prior stroke, Peripheral artery disease, Smoking, Alcohol, Antithrombotic therapy, SBP, Diastolic blood pressure, Platelet count, Blood coagulation tests, Glucose, LDL-c, Stroke subtype, NIHSS, Onset time.  In feature engineering, lesion topography and ASPECT score was computed based on CT scan.  No feature selection based on statistical significance criteria was performed. | Predictor variable importance calculation and prediction were compared among several machine learning models including: RBF, MLP, PNN and SVM | No |
| 5 | Efficiency | Three-month mRS | Thrombolysis | Only factors that had been shown to be significantly independently associated with outcome of ischemic stroke patients in previous studies were initially selected: CT scan, Prestroke mRS, Age, Glucose, Onset time, NIHSS.  In feature engineering, hyper dense cerebral artery sign and early infarct signs on admission CT scan was detected. Dummy variables were created for all features using different thresholds.  No feature selection based on statistical significance criteria was performed. | A simple risk score based on logistic regression coefficients | Yes, the scoring tool is based on baseline parameters and is quick to perform |
| 6 | Safety | Poststroke SICH | Thrombolysis | Only variables previously identified as factors associated with increased risk of Poststroke SICH were initially selected: Age, Gender, Onset time, NIHSS, CT scan, SBP, Diastolic blood pressure, Glucose, Blood coagulation tests, Platelet count, Leukocytes, Hypertension, Diabetes, Atrial fibrillation, hyperlipidemia, Other cardiac disease, Prior stroke, Antithrombotic therapy.  In feature engineering, hyperdense cerebral artery sign and early infarct sign on CT scan was detected. Dummy variables were created for all features using different thresholds.  Stepwise model building was performed to further select statistically significant features among dummy variables. | A simple risk score based on logistic regression coefficients | Yes, the score is based on easily assessable variables before administration of thrombolysis |
| 7 | Safety | Poststroke SICH | Thrombolysis | Only variables previously identified as factors associated with increased risk of Poststroke SICH were initially selected: Onset time, Age, Glucose, rtPA dose, Weight, NIHSS, SBP, Diastolic blood pressure, Gender, Hypertension, Diabetes, Hyperlipidemia, Smoking, Prior stroke, Atrial fibrillation, Other cardiac disease, Antithrombotic therapy, CT scan.  In feature engineering, early signs of infarct on CT scan was detected. Dummy variables were created based on all features using different thresholds and cutoff points in an exploratory manner.  Only variables showing an association with SICH at the $P\leq0.10$ level in the univariate analysis were included as potential predictors into the multivariate logistic regression model. | Risk score where the point values of each risk factors were based on their adjusted odd ratio in a multivariate logistic regression model | Yes, to be clinically practical, the risk score was designed to be easy to apply at the bedside using information available in the emergency situation |
| 8 | Safety | Poststroke SICH | Thrombolysis | Potential predictor variables were determined based on prior knowledge: Age, Gender, NIHSS, Race, SBP, Glucose, Blood coagulation tests, Creatinine, Antithrombotic therapy, Atrial fibrillation, Prior stroke, Other cardiac disease, Diabetes, Hypertension, Smoking, Hyperlipidemia.  In feature engineering, two interaction features were created: Antithrombotic therapy * Blood coagulation tests, Diabetes * Prior stroke.  Multivariable logistic regression was firstly used to identify independent predictors, then continuous covariates were evaluated for appropriateness of the linearity assumption using plots displaying the relationship of each variable with the log odds of SICH. Only variables showing a linear relationship with log odds of SICH throughout their range were included. | A simple risk score based on logistic regression coefficients | No |
| 9 | Safety and Efficiency | Poststroke SICH and a composite outcome at 3 months of mRS, NIHSS, Barthel index and Glasgow Outcome Scale score | Thrombolysis | The two most important prognostic factors for acute ischemic stroke were included: Age and NIHSS.  No feature engineering was performed.  No feature selection based on statistical significance criteria was performed. | SPAN index with high interpretability | Yes, the simple and practical index can be systematically and consistently applied in routine clinical practice |
| 10 | Safety | Poststroke SICH | Thrombolysis | Initially selected features: Age, NIHSS, Hypertension, Diabetes, Atrial fibrillation.    No feature engineering was performed.  No feature selection based on statistical significance criteria was performed. | THRIVE score with high interpretability | No |
| 11 | Safety | Poststroke SICH | Thrombolysis | Initially selected features: Age, Gender, Onset Time, NIHSS, SBP, Glucose, Blood coagulation tests, Platelet count, Antithrombotic therapy, CT scan.  In feature engineering, Acute ischemia extent, hyperdense MCA extent and white matter Fazekas score were calculated manually based on baseline CT scan. SICH-prognostic tools SEDAN and HAT scores were calculated using clinical and radiological input variables.  Significant variable were selected using logistic regression model. | SVM model without variable importance inference | No |
| 12 | Safety and Efficiency | Poststroke SICH and 3-month mRS | Thrombolysis and placebo | Initially selected features were based on prior related researches and discussions with six stroke neurologists. The predictors for the efficiency prediction were: Age, Gender, Prestroke mRS, NIHSS, Prior stroke, Hypertension, Diabetes, Hyperlipidemia, Atrial fibrillation, Antithrombotic therapy, SBP, Onset time, Stroke subtype. The predictors for the safety prediction were: Age, Gender, NIHSS, Hypertension, Diabetes, Hyperlipidemia, Antithrombotic therapy, SBP, Onset time, Stroke subtype.  In feature engineering, interaction terms between thrombolysis and other variables were created.  In feature selection, first a difference of -2 long-likelihood (-2LL) between models with and without the predictors was used to evaluate the effect of potential predictors. For the efficiency model, a fast backward elimination method using the Akaike information criterion (AIC) was then implemented further select significant features. For the safety model, the Lasso method was used to futher select significant features | Logistic regression with high interpretability | No |
| 13 | Efficiency | 3-month mRS | Thrombolysis and placebo | Initially selected features were based on previous researches: Age, Gender, Diabetes, Prior stroke, SBP, Glucose, Onset time, NIHSS  In feature engineering, the authored identified two variables that had been known to modify the effectiveness of thrombolysis: Onset time and SBP; and created interaction terms using thrombolysis treatment and these two variables.  Variable selection relied on the previously published researches with no additional data exploration using statistical method. | Logistic regression with high interpretability | Yes, the model was designed to be simple to be used in the emergency department. To simplify the implementation of the model, the authors explored an 8-item and a 3-item NIHSS to replace the 15-item NIHSS. Since ASPECT score was difficult to calculated in emergency, this CT feature was excluded |
| 14 | Safety | Poststroke SICH | Thrombolysis | Initially selected features were: Gender, Age, Diabetes, Glucose, Hypertension, Hyperlipidemia, Other cardiac disease, Atrial fibrillation, Prior stroke, Antithrombotic therapy, Pulse rate, SBP, Diastolic blood pressure, NIHSS, Platelet count, Blood coagulation tests, Creatinine, Onset time.  No feature engineering was performed.  Parameters with a $P\leq0.05$ were entered in a univariate regression equation to yield an ordinal odd ratio; parameters with high odd ratio and low P values were selected for further multivariable analysis. | Logistic regression with high interpretability | No |
| 15 | Safety | Poststroke SICH | Thrombolysis | Initially selected features were: Age, Hypertension, Diabetes, Hyperlipidemia, Smoking, Atrial fibrillation, Other cardiac disease, Antithrombotic therapy, Prestroke mRS, NIHSS, CT scan, SBP, Glucose, Onset time.  In feature engineering, hyperdense artery sign and current infarction sign was computed on CT scan.  A multivariate logistic regression analysis was firstly performed using a forward stepwise method that included all variables with a probability value <0.1 in the univariate analysis; parameters of logistic regression with high odd ratio and low P values were selected for composing the nomogram. | A nomogram with high interpretability | No |
| 16 | Efficiency | A combined score of mRS and NIHSS as an early clinical outcome assessment; A long-term 3-month mRS | Thrombolysis and placebo | Initially selected features were: Age, Gender, Onset time, SBP, Diastolic blood pressure, Hypertension, Diabetes, Hyperlipidemia, Atrial fibrillation, Prior stroke, DWI, PWI.  In feature engineering, global infarct core volume, penumbra volume and mismatch between infarct core volume and penumbra volume was calculated. The mean ADC and mean CBF were then quantified in infarct core and penumbra. The brain was then divided into six gray matter regions plus a white matter region; In each region, regional radiological features were calculated (infarct core ratio, penumbra ratio, mean ADC, mean CBF).  The LASSO was conducted to select the most predictive features for clinical outcome. | Logistic regression with high interpretability | No |
| 17 | Efficiency | 3-month mRS | Thrombolysis | Four strong predictors were identified by three neurologists with clinical expertise: NIHSS, Age, Prestroke mRS, Onset time.  No feature engineering was performed.  No feature selection based on statistical significance criteria was performed. | A nomogram with high interpretability | Yes, the model was developed based on clinical variables easily available when thrombolysis starts |
| 18 | Efficiency | 24-hour NIHSS and 3-month mRS | Thrombolysis | Initially selected features were: Age, Gender, Onset time, NIHSS, SBP, Glucose, Temperature, Hypertension, Diabetes, Hyperlipidemia, Atrial fibrillation, CT.  No feature engineering was performed.  No feature selection based on statistical significance criteria was performed. | A black box CNN | No |
| 19 | Safety | Poststroke SICH | Thrombolysis | Initially selected features were: Age, Gender, Glucose, SBP, Diastolic blood pressure, CT, LDL-c, Hb A1C, Antithrombotic therapy, Blood coagulation tests, NIHSS, Onset time, Weight, Platelet count, Atrial fibrillation.  Presence of early infarct signs were computed from CT scan.  Parameters with a $P\leq0.05$ were considered statistically significant . | A binary logistic regression with high interpretability | No |
| 20 | Safety | Poststroke SICH | Thrombolysis | Initially selected features were: Age, Gender, Onset time, NIHSS, SBP, Glucose, Hypertension, Diabetes, Hyperlipidemia, Atrial fibrillation, Prior stroke, Other cardiac disease, Antithrombotic therapy, Smoking, Alcohol, Diastolic blood pressure.  No feature engineering was performed.  Wrapper method, correlation-based feature selection and conservative-mean method were used to select significant features. | Performances were compared among logistic regression, neural network, SVM, random forest and AdaBoost, without any model inference analysis | No |
| 21 | Safety | Poststroke SICH | Thrombolysis | Initially selected features were: Age, Gender, Onset time, BMI, Glasgow coma scale score, SBP, Diastolic blood pressure, NIHSS, Stroke subtype, Hypertension, Diabetes, Hyperlipidemia, Atrial fibrillation, Prior stroke, Other cardiac disease, Smoking, Glucose, Creatinine, LDL-c, Hb A1C.  No feature engineering was performed.  One-way ANOVA was used for continuous variables, and Fisher’s exact test was used for categorical variables to select significant features. | A neural network AUNet was built for prediction, without any model inference analysis | No |
| 22 | Efficiency | 3-month mRS | Thrombolysis | Initially selected features were: Age, Gender, Smoking, Hypertension, Diabetes, Prior stroke, Hyperlipidemia, Antithrombotic therapy, Hyperhomocysteinemia, Atrial fibrillation, NIHSS, SBP, Diastolic blood pressure, Onset time, Glucose, Leukocytes, Blood coagulation tests, LDL-c, Uric acid.  No feature engineering was performed.  Variables with a $P\leq0.1$ in the univariate analysis were included in multivariate logistic regression analysis using a backward selection method. The variables with a $P\leq0.05$ in the binary logistic regression analysis were considered statistically significant. | A nomogram with high interpretability | No |
| 23 | Safety | Poststroke SICH | Thrombolysis | Initially selected features were: MRI, Age, Gender, SBP, Diastolic blood pressure, Glucose, Prior stroke, Smoking, Hyperlipidemia, Antithrombotic therapy, Hypertension, Diabetes, Atrial fibrillation, NIHSS, Onset time, Platelet count, LDL-c, Creatinine, C-reactive protein, Uric acid.  In feature engineering, the chronic disease scale was rated based on hypertension, diabetes and atrial fibrillation. Cerebral small vessel disease score was computed from MRI sequences.  A multivariable logistic regression was built to identify statistically significant variables. | A nomogram with high interpretability | No |
| 24 | Safety | Poststroke SICH | Thrombolysis | Initially selected features were: Age, Gender, Smoking, BMI, Diabetes, Atrial fibrillation, Hypertension, Glucose, Hyperlipidemia, LDL-c, Platelet count, SBP, Diastolic blood pressure, NIHSS, Onset time, Antithrombotic therapy.  No feature engineering was performed.  A multivariable logistic regression analysis was performed to predict the probability of SICH using a forward stepwise method that included all variables with a $P\leq0.2$ in the univariable analysis. Variables with a $P\leq0.05$ in the multivariable logistic regression were entered into the prediction model. | A nomogram with high interpretability | No |
| 25 | Efficiency | Final infarct volumes for acute ischemic stroke patients with full or no recanalization | Thrombolysis | Initially selected features were: DWI and ADC.  No feature engineering was performed.  No feature selection based on statistical significance criteria was performed. | Blackbox combining an adaptive linear ensemble model and a deep U-Net | No |
| 26 | Efficiency | 6-month mRS | Thrombolysis | Initially selected features were: Age, Gender, Stroke subtype, Smoking, Alcohol, NIHSS, Antithrombotic therapy, Hypertension, Diabetes, Hyperlipidemia and Atrial fibrillation  No feature engineering was performed.  Variables with $P\leq0.05$ in univariate logistic regression analysis were included in the multivariate logistic regression model, and the step-wise regression method was used for variable selection. | A nomogram with high interpretability | No |
| 27 | Efficiency | mRS at discharge; 3-month mRS and mortality | Thrombolysis and placebo | Initially selected features were: Age, Gender, NIHSS, Hypertension, Diabetes, Prior stroke, Hyperlipidemia, Atrial fibrillation, Smoking, , Other cardiac disease  No feature engineering was performed.  One-way ANOVA was used for continuous variables, and Fisher’s exact test was used for categorical variables to select significant features. | Performances were compared among Logistic regression, Artificial neural network, Naïve Bayes classifier and gradient boosting decision tree, without any model inference analysis | No |
| 28 | Safety | Poststroke SICH | Thrombolysis | Initially selected features were: NIHSS, Age, Pulse pressure, Prestroke mRS, SBP, Glucose, Diastolic blood pressure, Weight, Onset time, Hypertension, Antithrombotic therapy, Smoking, Hyperlipidemia, Atrial Fibrillation, Other cardiac disease, Diabetes, Prior stroke, Gender.  In feature engineering, continuous variables were stratified into categorical variables using k-means clustering with the cluster boundary optimized for maximum SICH risk.  Odds-ratios were computed for all categorical variables to identify those with a stronger association with SICH ($P\leq0.2$). Categorical variables having odds-ratios higher than 1.5 were included into the final model. | A risk score based on odds-ratios with high interpretability | Yes, SICH risk scoring models should not be computationally intensive and features should be readily available in the hyperacute clinical stroke setting |
| 29 | Efficiency | 1-hour NIHSS | Thrombolysis | Initially selected features were: Age, BMI, Gender, Prior stroke, NIHSS, Other cardiac disease, Blood coagulation tests, Red cell distribution width test, Leukocytes, Myoglobin, Glucose  No feature engineering was performed.  Only features with a statistical significance of $P\leq0.05$ were included into the final model. | Performances were compared among logistic regression and tree-based machine learning algorithms. The recursive feature elimination method was used to calculate the contribution of each of the variables. | No |

Note: SBP = Systolic blood pressure; DWI = Diffusion-weighted imaging; PWI = Perfusion-weighted imaging; ADC = Apparent diffusion coefficient; CBF = Cerebral blood flow; mRS = Modified Rankin Scale; NIHSS = National Institutes of Health Stroke Scale; CT = Computed tomography; MCA = Middle cerebral artery; SICH = Symptomatic intracerebral hemorrhage; SVM = Support vector machine; LDL-c: Low-density-lipoprotein cholesterol; ASPECT = Alberta stroke programme early CT score; RBF = Radial basis function; MLP = Multilayer perception; PNN = Probabilistic neural network; rtPA = Recombinant tissue plasminogen activator; LASSO = Least Absolute Shrinkage and Selection Operator; CNN = Convolutional Neural Network; Hb A1C = Hemoglobin A1c; TICI = Thrombolysis in cerebral infarction; BMI = Body mass index; MRI = Magnetic resonance imaging
